# Excellent Intra and Inter-Observer Reproducibility of Wrist Circumference Measurements in Obese Children and Adolescents

**DOI:** 10.1371/journal.pone.0156646

**Published:** 2016-06-13

**Authors:** Giuseppe Campagna, Simona Zampetti, Alessia Gallozzi, Sara Giansanti, Claudio Chiesa, Lucia Pacifico, Raffaella Buzzetti

**Affiliations:** 1 Department of Experimental Medicine, Sapienza University, Rome, Italy; 2 Policlinico Umberto I Hospital, Sapienza University, Rome, Italy; 3 Institute of Translational Pharmacology, National Research Council, Rome, Italy; Medical Clinic, University Hospital Tuebingen, GERMANY

## Abstract

In a previous study, we found that wrist circumference, in particular its bone component, was associated with insulin resistance in a population of overweight/obese children. The aim of the present study was to evaluate the intra- and inter-operator variability in wrist circumference measurement in a population of obese children and adolescents. One hundred and two (54 male and 48 female) obese children and adolescents were consecutively enrolled. In all subjects wrist circumferences were measured by two different operators two times to assess intra- and inter-operator variability. Statistical analysis was performed using SAS v.9.4 and JMP v.12. Measurements of wrist circumference showed excellent inter-operator reliability with Intra class Correlation Coefficients (ICC) of 0.96 and ICC of 0.97 for the first and the second measurement, respectively. The intra-operator reliability was, also, very strong with a Concordance Correlation Coefficient (CCC) of 0.98 for both operators. The high reproducibility demonstrated in our results suggests that wrist circumference measurement, being safe, non-invasive and repeatable can be easily used in out-patient settings to identify youths with increased risk of insulin-resistance. This can avoid testing the entire population of overweight/obese children for insulin resistance parameters.

## Introduction

Obesity is the major risk factor for the development of insulin resistance in children and adolescents [1]. Insulin resistance is believed to play a central role in the pathogenesis of cardiovascular disease (CVD), and its prevalence in the pediatric population is increasing, particularly among obese children and adolescents [2].

In clinical practice, anthropometric measurements such as BMI, waist circumference, waist to height ratio are quick and non-invasive tools for evaluating body adiposity [3]. Currently, BMI is the most common measurement to evaluate overweight and obesity in children and adolescents, however it is not accurate enough to determine total body fat and its distribution. Moreover, BMI is not only related to body fat but also to fat-free mass, and this is especially relevant during normal growth in children [3]. Waist circumference, also, presents some limitations such as the lack of uniformly accepted measurement protocol [4, 5]. Furthermore, waist circumference measurement is subject to significant inter-operator variability [6]. Waist to height-ratio has been introduced as a suitable alternative measure for assessing central fatness in children, since it is relatively age-independent and that in normalizing for growth, it might obviate the need for age-related reference charts [7, 8]. However its independence of ethnic and age is arguable [9]. Moreover waist to height-ratio is dependent on waist circumference.

In the light of the limitations of the common anthropometric measures, in a previous study we identified a new anthropometric marker of insulin resistance in children and adolescents: the wrist circumference [10]. We found that wrist circumference, in particular its bone component, is highly correlated with measures of insulin-resistance in a population of overweight/obese children and adolescents. Wrist circumference is an easy-to-detect measure of skeletal frame size [11–13] and it is no strictly confounded by body fat variation [14].

To the best of our knowledge, there has been no previous study analyzing the reproducibility in measuring wrist circumference performed by clinical operators.

In view of this the aim of the present study was to evaluate the intra- and inter-operator variability in measuring wrist circumference in a population of obese children and adolescents.

## Materials and Methods

One hundred and two (54 males and 48 females, mean age 9.8 ± 2.9, range age: 3–16 years) obese children and adolescents were consecutively enrolled by Department of Pediatrics, “Sapienza” University of Rome. The study protocol was approved by the Ethical Committee of the “Sapienza” University of Rome, and parents gave written consent for their children to participate in the study after being informed of its nature.

In all children and adolescents, fasting glucose, fasting insulin levels, and lipid profiles were evaluated at entry. Serum total cholesterol, high-density lipoprotein cholesterol, and triglyceride levels were determined by a Technicon RA-1000 Autoanalyzer. Glucose levels were determined by the glucose oxidase method (Autoanalyzer, Beckman Coulter, Brea, CA). Serum insulin was measured by radioimmunoassay (Adaltis Insulin Kit, Bologna, Italy). Insulin resistance was estimated according to the homeostasis model assessment of insulin resistance (HOMA-IR).

Waist circumference was measured with a flexible tape at the level of the umbilicus, and was recorded to the nearest millimeter. The blood pressure measurement followed the recommendation of the Fourth Report on the Diagnosis, Evaluation and Treatment of High Blood Pressure in Children and Adolescents. Blood pressure was measured while children were sitting and with the cubital fossa supported at heart level, after at least 5 min of rest. Blood pressure were measured using a mercury sphygmomanometer, with the appropriate cut off for the children upper arm size.

Dominant wrist circumference was measured with subjects in a seated position using a tension-gated tape measure positioned over the Lister tubercle of the distal radius and over the distal ulna [15]. The Lister tubercle, a dorsal tubercle of the radius, can be easily palpated [16] at the dorsal aspect of the radius around the level of the ulna head, [17] about 1 cm proximal to the radio carpal joint space [18]. A tension-gated tape measure was used to ensure equivalent tape pressure between subjects (S1 Fig).

The wrist circumferences were measured by two different operators (two medical doctors: AG and SG) two times to assess intra- and inter-operator variability (S1 Database). The two operators were in two different rooms; indeed, while one operator was taking measurements the other was not observing (S2 Fig). The same measuring tape was used throughout this study.

### Statistical Analysis

Statistical analysis was performed using SAS v. 9.4 and JMP v. 12 (SAS and JMP, Institute Inc., Cary, NY, USA). Variables were tested for normality by the Shapiro-Wilk test. The repeated measurements were plotted against each other and if the measurements were like then all points lie on the same straight line. Berg plot is useful to graphically present and assess comparisons between the measurements of two different operators. The Berg plot shows a two dimensional scatter plot in the form of a box-whisker plot on two variables as well as on their differences [19]. The Intra class Correlation Coefficients (ICC) for agreement were used to test for reproducibility of the wrist circumference measurements among the two operators. The ICC is a measure of the amount of overall data variance due to between-subjects variability. ICC emphasizes the ‘‘interchangeability” of the operators [20–21]. It is been calculated with the macro %ICC.

$$ICC=\frac{\left(\sigma_{\alpha }^{2}-\sigma_{\gamma }^{2}\right)}{j-1}*\frac{1}{\sigma_{\alpha }^{2}-s_{\beta }^{2}-\sigma_{\gamma }^{2}-\sigma_{e}^{2}}$$

- $\sigma_{\alpha }^{2}=Var_{i}(\alpha_{i})$: is the random effect variance of subject *i*
- $s_{\beta }^{2}=\frac{\sum_{j=1}^{n}{\left(\beta_{j}-\bar{\beta }\right)}^{2}}{n-1}$: is the fixed effect variance of operator *j*
- $\sigma_{\gamma }^{2}=Var_{i}(\gamma_{ij})$: is the random interaction effect variance
- $\sigma_{e}^{2}=Var(e_{ijk}|i,j)$: is the random error component variance

Let α_ι_ is the random effect of subject i, β_j_ is fixed effect of operator j, $\bar{\beta }$ is fixed effect mean of operator j, γ_ij_ is random interaction effect, e_ijk_ is random error component and n is number of operators.

The Bland and Altman [22] plot showed the agreement among the measurements.

The Concordance Correlation Coefficient (CCC) is based on the distance in the plane of each pair of data to the 45 degree line through the origin and was used to quantify the reproducibility of multiple readings made by same operator [23]. It is been calculated with macro %CCC.

$$CCC=\frac{2\rho \sigma_{y}\sigma_{x}}{{\left(\mu_{y}-\mu_{x}\right)}^{2}+\sigma_{y}^{2}+\sigma_{x}^{2}}$$

- *ρ* = *corr*(*x*_*ij*_,*y*_*ij*_): correlation for *i*^*th*^ subject and *j*^*th*^ operator
- *μ*_*y*_ = *mean*(*y*_*ij*_)
- *μ*_*x*_ = *mean*(*x*_*ij*_)
- $\sigma_{y}=\sqrt{Var(y_{ij})}$: standard deviation
- $\sigma_{x}=\sqrt{Var(x_{ij})}$: standard deviation

A paired Student’s test was used to detect significant intra- and inter-operator differences in the quantitative measurements.

## Results

The clinical and biochemical parameters of the 102 obese children are shown in Table 1.

**Table 1:** Clinical and anthropometric characteristics of 102 children and adolescents.

	Mean ± SD
n (Male/Female)	102 (54/48)
Age (years)	9.8 ± 2.8
BMI z-score	2.4 ± 0.5
Wrist circumference (cm)	16.1 ± 1.3
Waist circumferences (cm)	85.6 ± 8.7
Systolic blood pressure (mmHg)	112.7 ± 10.7
Diastolic blood pressure (mmHg)	65.1 ± 8.6
Total cholesterol (mg/dL)	157.4 ± 34.0
HDL-cholesterol (mg/dL)	51.5 ± 13.6
LDL-cholesterol (mg/dL)	89.6 ± 29.7
Triglycerides (mg/dL)	79.1 ± 44.2
Fasting insulin (μU/mL)	13.5 ± 9.3
Fasting glucose (mg/dL)	83.2 ± 6.1
HOMA-IR	2.8 ± 2.1

We observed that there were no statistically significant differences between the means of the first and the second wrist circumference measurements performed by the same operators (p = 0.33 and p = 0.23) and between the two different operators (p = 0.17 and p = 0.37).

**Fig 1:**
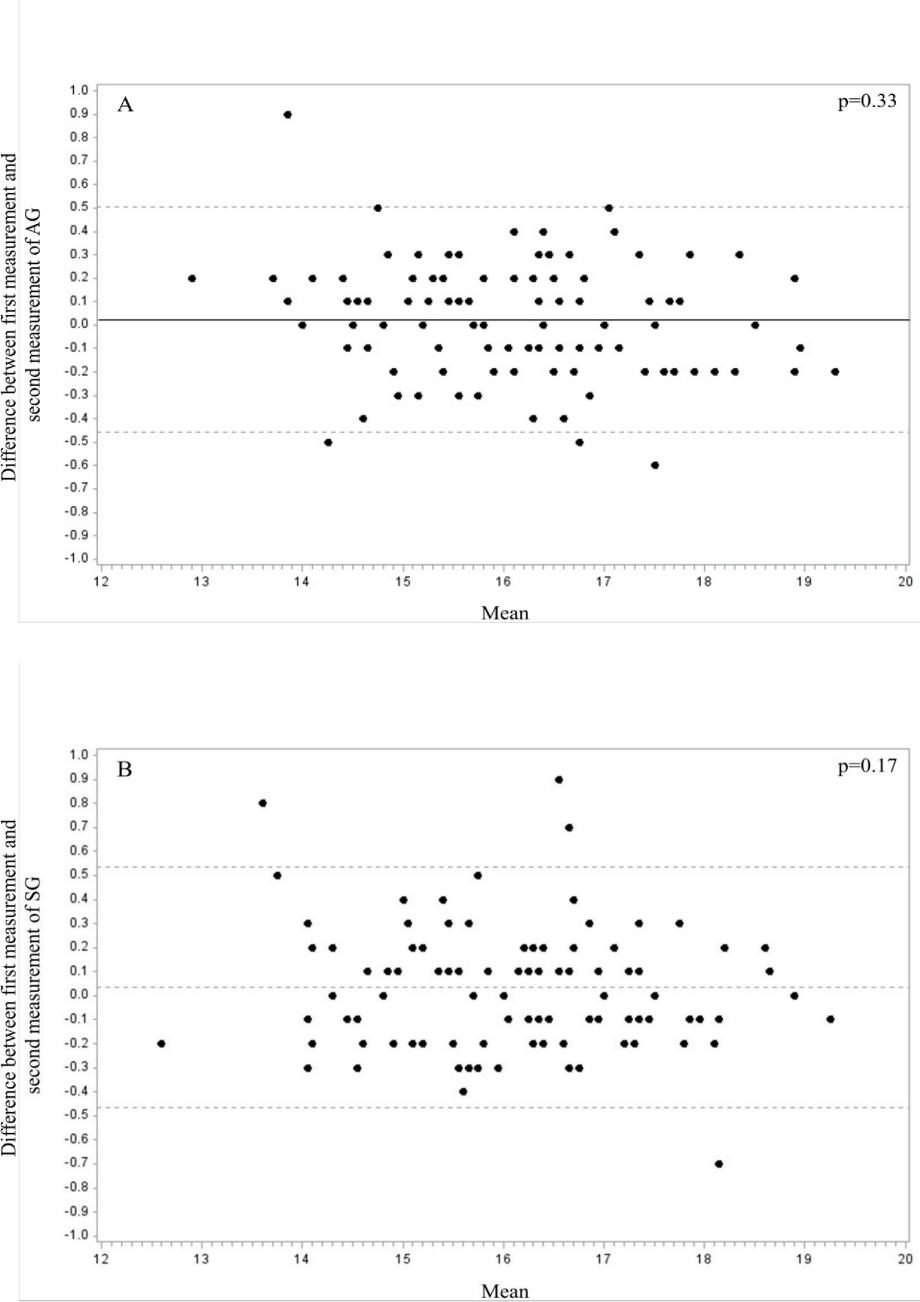
Bland and Altman plots for the first and second wrist circumference measurement performed by the two operators.

Measurements of wrist circumference showed excellent inter-operator reliability with ICC of 0.96 (0.94–0.98) and ICC of 0.97 (0.95–0.98) for the first and the second measurement, respectively. The intra-operator reliability was also very strong with a CCC of 0.98 (0.98–0.99) and of 0.98 (0.97–0.99) for the first and second operator, respectively. Bland-Altman plots were used to display the variability in repeat measurements of wrist circumference by each operator. Fig 1A shows the plot for operator A and Fig 1B for operator B. We found that only 4.1% of all the measurements done by two operators fell outside the 95% agreement limits; indicating a high degree of agreement among repeated measurements.

**Fig 2:**
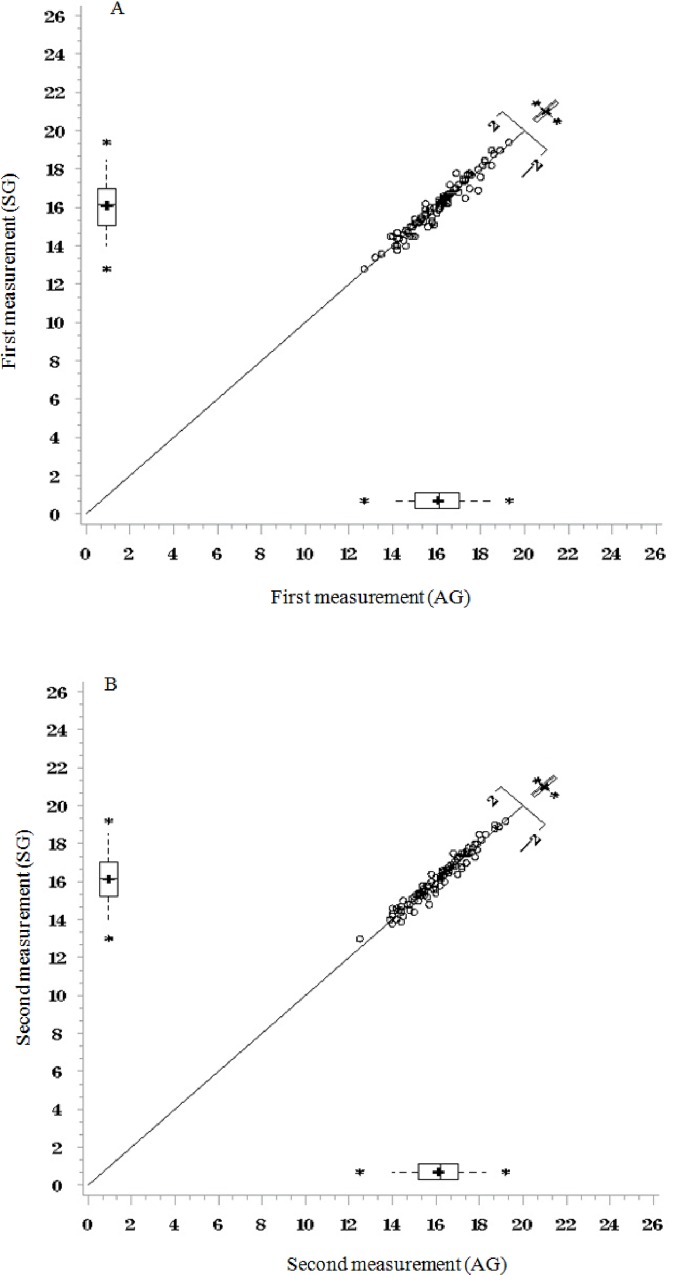
Berg plots for first and second wrist circumference measurement performed by the two operators.

Berg plots show the inter-operator agreement for the first and the second wrist circumference measurements, respectively (Fig 2A and 2B). All two plots show a good distribution of data points around the 45° degree line, indicating a high degree of correlation among the wrist circumference measurements performed by the two operators. The box-plots reveal symmetry in data distributions indicating normality (Fig 2A and 2B).

## Discussion

The results of the present study suggest an excellent intra- and inter-operator reliability of wrist circumference measurements in obese children and adolescents. In particular, the very high values of ICC and CCC (both≥0.96) indicate almost perfect inter—and intra-observer reproducibility of wrist circumference measurements. Agreement that is less than 100% may be due to errors in positioning, recording or transcribing of wrist circumference measurements.

Our findings underline that a single operator measurement is most likely satisfactory to evaluate wrist circumference in children and adolescents.

Measurement of wrist circumference can provide as an easy-to-detect clinical marker of insulin resistance to identify young subjects at increased risk of CVD [10]. The wrist circumference parameter is easily obtainable and measurable by the operator, reducing the cooperation needed by the subjects [10]. The easy and simple way of measuring wrist circumference is a considerable advantage in everyday clinical practice compared to the classical anthropometrical measurement such as BMI or waist circumference. Another advantage of using wrist circumference compared to the common anthropometric measures is that reflecting a skeletal frame size, it changes slightly through time [24].

Differently from waist circumference, wrist circumference measurement is not affected by clothing, respiration or by postprandial state which can interfere with the determination of waist circumference [24]. To date, there is no agreement on the optimal anatomical sites to measure waist circumference in children [25]. When waist circumference measurements were performed below the last rib, the values were the lowest, while the maximum values were observed above the iliac crest [26]. Another study, reported that waist circumference measurements performed on the umbilicus showed statistically higher values in females compared to males [27]. These findings highlight that comparing results regarding waist circumference measurements that adopted various anatomical sites in different studies is not suitable [27].

Recently, neck circumference has been identified as a new anthropometric parameter that could be used to screen for overweight and obesity in children [28].

LaBerge *et al* found that neck circumference showed very good inter and good intra-rater reliability in children 6–16 years old [29]. Multiple measurements are not required for precision and reliability.

Neck circumference measurement is simple, and can easily be learned and applied in general practice by medical doctors [30]. Additional studies to assess the usefulness of neck circumference as a marker of obesity are needed [31]. Moreover, we hypothesize that neck circumference being connected with skeletal elements of the shoulder girdle should track along with overall muscle mass and thus it could be influenced by physical activity.

In 2011, we found that wrist circumference was highly correlated with measures of insulin resistance in a population of n = 477 overweight/obese children and adolescents [10]. Following our study, other Authors reported a significant association between wrist circumference and insulin resistance both in children and in adults. In a sample of non-Hispanic white youth aged 8–18 years, wrist breadth was associated with HOMA-IR independent of adiposity measures [32]. The correlation between wrist breadth and HOMA-IR appeared to be stronger than other frame size measures after adjusting for objectively measured total body fat. In another cross-sectional and 8.8 year follow up study, performed in adult population, including 6393 subjects (3677 females, 2716 males) without prevalent diabetes, wrist circumference was found to be a significant predictor of diabetes and metabolic syndrome [33]. The results of all these studies highlight that wrist circumference could be a new interesting anthropometric marker for the identification of subjects developing diabetes, metabolic syndrome and CVD.

To our knowledge, this is the first study reported in literature to investigate the reproducibility in measuring the wrist circumference performed by two different operators. The originality and the most striking finding of this study is the demonstration that wrist circumference measurement has excellent repeatability and reproducibility and represents an anthropometric parameter easily to measure by any operator. Limitations of our study could apparently be due to the small sample size, limited number of the measurements determined and performed by two operators only, but, on the other hand, these restrictions are offset by the excellent degree of reproducibility (both inter- and intra-operator variability) obtained from our results. Regarding variability in waist circumference, some studies revealed a significant inter- observer variability in its measurements [34]. As reported by Panoulas *et al* written instructions as a form of training, may eliminate the systematic error but, does not reduce the overall variation in waist circumference measurements [6]. A study performed on neck circumference to evaluate inter- and intra-rater reliability of measurements in children (divided in two groups: 6–10 and 11–16) found excellent results (ICC>0.9) but in a very small sample size (18 and 40 children) [29].

These results underline that almost all studies performed to assess the reliability of the common anthropometric measures have some limitations due to a significant variability between operators or limited number of patients analyzed.

In conclusion, the high reproducibility demonstrated in our results suggests that wrist circumference measurement, being inexpensive, safe, non-invasive and highly repeatable, might be a valid method to predict insulin resistance in both obese children and adolescents.

## Supporting Information

S1 Database (XLS)

S1 Fig Example of wrist circumference measurement performed by an operator. (TIF)

S2 Fig Flow chart of study design. (TIF)
