# Supplementary figures and images for: Excellent Intra and Inter-Observer Reproducibility of Wrist Circumference Measurements in Obese Children and Adolescents
Source: PLoS One. 2016 Jun 13;11(6):e0156646. doi: 10.1371/journal.pone.0156646 (PMC4905645; doi:10.1371/journal.pone.0156646)

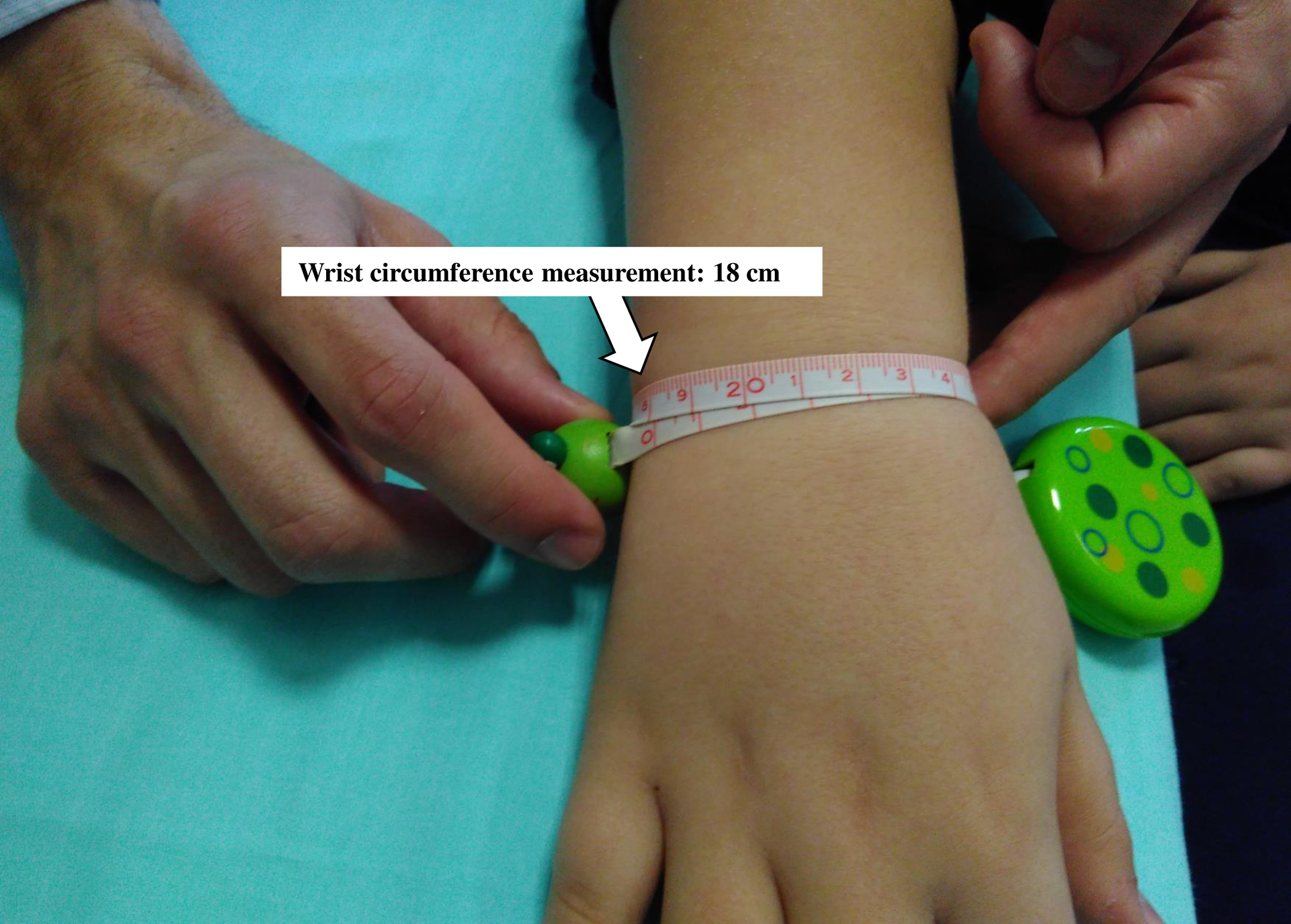

Supplement: S1 Fig — (TIF) [file pone.0156646.s002.tif]

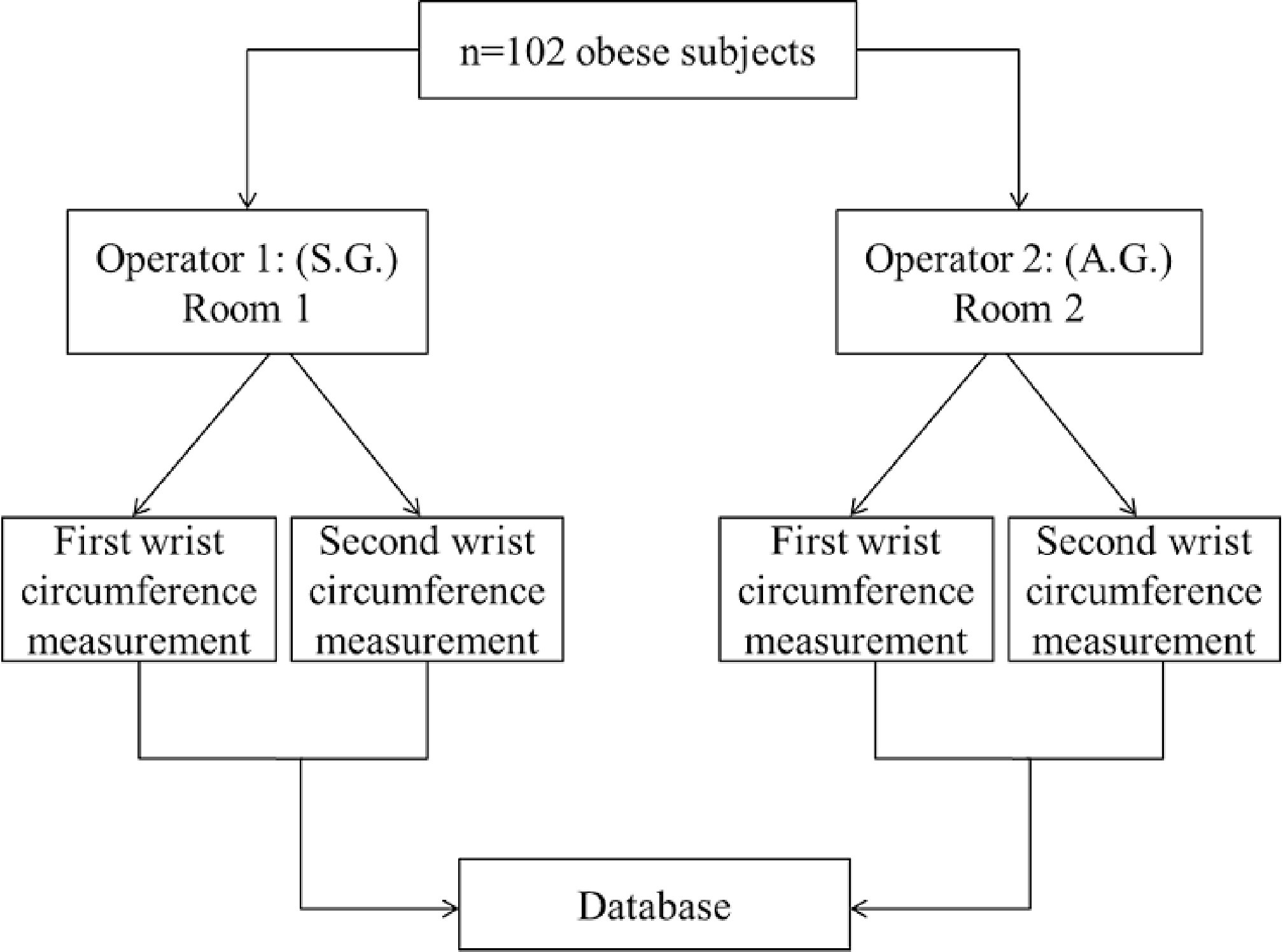

Supplement: S2 Fig — (TIF) [file pone.0156646.s003.tif]
